# Optimized Microfluidic Biosensor for Sensitive C-Reactive Protein Detection

**DOI:** 10.3390/bios15040214

**Published:** 2025-03-26

**Authors:** Amirmahdi Tavakolidakhrabadi, Matt Stark, Alexander Küenzi, Sandro Carrara, Cédric Bessire

**Affiliations:** 1 Institute for Human Centered Engineering HuCE, Department of Engineering and Computer Science, Bern University of Applied Sciences, 2501 Biel, Switzerland; amirmahdi.tavakolidakhrabadi@bfh.ch (A.T.);; 2 Bio/CMOS Interfaces Laboratory (BCI), École Polytechnique Fédérale de Lausanne (EPFL), 2002 Lausanne, Switzerland

**Keywords:** conjugated fluorescent antibodies, gold nanoparticle-based detection, microfluidic integration, extended detection range for CRP measurement, microfluidic-controlled LFIA for CRP

## Abstract

Lateral flow immunoassays (LFIAs) were integrated into microfluidic chips and tested to enhance point-of-care testing (POCT), with the aim of improving sensitivity and expanding the range of CRP detection. The microfluidic approach improves upon traditional methods by precisely controlling fluid speed, thus enhancing sensitivity and accuracy in CRP measurements. The microfluidic approach also enables a one-step detection system, eliminating the need for buffer solution steps and reducing the nitrocellulose (NC) pad area to just the detection test line. This approach minimizes the non-specific binding of conjugated antibodies to unwanted areas of the NC pad, eliminating the need to block those areas, which enhances the sensitivity of detection. The gold nanoparticle method detects CRP in the high-sensitivity range of 1–10 μg/mL, which is suitable for chronic disease monitoring. To broaden the CRP detection range, including infection levels beyond 10 μg/mL, fluorescent labels were introduced, extending the measuring range from 1 to 70 μg/mL. Experimental results demonstrate that integrating microfluidic technology significantly enhances operational efficiency by precisely controlling the flow rate and optimizing the mixing efficiency while reducing fabrication resources by eliminating the need for separate pads, making these methods suitable for resource-limited settings. Microfluidics also provides greater control over fluid dynamics compared to traditional LFIA methods, which contributes to enhanced detection sensitivity even with lower sample volumes and no buffer solution, helping to enhance the usability of POCT. These findings highlight the potential to develop accessible, accurate, and cost-effective diagnostic tools essential for timely medical interventions at the POC.

## 1. Introduction

Current diagnostic methods such as ELISAs (Enzyme-Linked Immunosorbent Assays) and traditional LFIAs exhibit several limitations that impact their clinical relevance and applicability. While highly sensitive and specific, ELISAs require complex laboratory infrastructure, skilled personnel, and long processing times, making it less suitable for POC applications. Traditional LFIA, on the other hand, offers rapid results and ease of use but suffers from lower sensitivity and semi-quantitative outputs, limiting its reliability in detecting low-abundance biomarkers. This research aims to address the challenges associated with LFIAs.

The medical field has long depended on key biomarkers such as CRP to evaluate infection levels [1]. CRP is an acute-phase biomarker synthesized by the liver in response to inflammatory stimuli [2]. Widely utilized in clinical settings, CRP serves as a crucial indicator for assessing the presence and severity of infection or systemic inflammation [3]. Its serum concentrations rise significantly during inflammatory processes, providing a rapid, albeit non-specific, measure of immune activation [4]. This characteristic makes CRP testing an essential tool for clinicians to monitor and evaluate the body’s inflammatory response to infectious agents [5].

CRP levels, at low concentrations (1–5 μg/mL), are indicative of cardiovascular risk [6], while elevated levels (>10 μg/mL) can distinguish between bacterial infections (>20 μg/mL) and viral infections (<20 μg/mL) [7]. CRP is especially vital for POCT, which requires rapid and accurate diagnostics [8]. However, the variability of these biomarkers based on the type and stage of infection has posed challenges in diagnostic accuracy [9]. To address this, a novel microfluidic platform has been developed to measure essential diagnostic markers like CRP, which plays a critical role in the body’s inflammatory response.

Numerous LFIAs are commercially available, capable of providing either qualitative or quantitative biomarker measurements [10]. LFIA technology has evolved from its origins in paper-based assays, which were among the first POC diagnostics developed due to their simplicity and low cost. These traditional paper-based systems, however, have inherent limitations, including restricted sensitivity, limited quantitative capability, and a lack of control over fluid dynamics, which often leads to variability in results.

Blocking strategies are essential for minimizing non-specific binding, which can affect LFIA sensitivity and accuracy. Various agents, such as BSA, casein, and PEG, have been evaluated to optimize signal-to-noise ratios. In this study, a comparative analysis of blocking agents was conducted, revealing that a dual-blocking approach significantly reduced background noise and improved assay reproducibility.

In conventional LFIAs, the testing process involves two primary steps. Initially, a drop of the sample solution is applied to the sample pad, followed by the addition of a buffer solution to facilitate the migration of the sample across the NC membrane [11]. The buffer not only drives the fluid flow but also plays a critical role in preventing the non-specific binding of antibodies [12], which could otherwise lead to inaccuracies by adhering to unintended regions of the pad rather than the designated test line. Conventional LFIAs often require relatively large sample volumes and multiple handling steps, such as adding buffer and sample separately, which increases the complexity and potential for human error. These issues, along with limited sensitivity and difficulties in accurately quantifying results, are significant.

In this study, a streamlined method was developed that involves pre-blocking the NC pad, which significantly simplifies the assay procedure. This pre-blocking approach reduces the need for separate buffer addition, resulting in a single-step process for the accurate detection of CRP concentrations. This method not only minimizes the fabrication complexity but also reduces the required volume of reagents and simplifies the overall workflow. Typically, a conjugate pad is used to immobilize CRP antibodies conjugated with fluorescent labels. To eliminate the need for a separate conjugate pad, the designed labeled structure serves as a substrate where a drop of antibody solution containing the fluorescent label can be directly applied and allowed to dry. This structure significantly enhances the available surface area for drying in microfluidics. The integration of microfluidic technology obviates the requirement for supplementary fluid dispensing apparatus by incorporating the functionality of a conjugate pad [13]. This innovation streamlines the assay preparation process, thereby reducing overall costs and minimizing technical complexity. Furthermore, the extended reaction time provided by this design enhances binding interactions, leading to the increased reaction efficiency and, ultimately, improved sensitivity of the detection system.

A major issue is the lack of precise fluid flow control due to capillary-driven movement on the NC pad, leading to variability and inconsistent reagent distribution, which affects detection reliability. Integrating LFIAs into microfluidics offers precise fluid control, reduced reagent use, and enhanced sensitivity through optimized handling. Microfluidics ensures the accurate manipulation of small fluid volumes, reducing waste and improving test reproducibility. It also minimizes variability and ensures the uniform distribution of CRP antibodies, enhancing both the sensitivity and accuracy of quantitative analysis.

The centerpiece of this platform is a CRP biosensor that employs a clinically validated nitrocellulose paper strip. Various types of labels, such as fluorescent markers and gold nanoparticles, are used to determine which types of labels can enhance the sensitivity and expand the detection range. The color intensity of the band on the strip directly correlates with CRP levels, providing a clear, quantifiable visual indicator of this important biomarker [14].

Additionally, the differences and limitations of CRP detection using gold nanoparticles and fluorescent labels are thoroughly investigated. The goal of this study was to identify the most effective labeling technique for enhancing the sensitivity and expanding the detection range of CRP, specifically to cover the full CRP range necessary for distinguishing not only increased inflammatory levels, but also distinguishing between viral and bacterial infection levels. Various labeling techniques were assessed in terms of their sensitivity and overall effectiveness. Ultimately, the most suitable labeling method was selected for its superior sensitivity in measuring CRP concentrations and its ability to accurately detect a broader range of CRP levels, thereby supporting more reliable inflammatory and infection level monitoring. This approach aims to enhance diagnostic accuracy and reliability across a wide array of clinical settings.

## 2. Materials and Methods

### 2.1. CRP Detection Using Fluorescent and Gold Nanoparticle Labels

LFIAs are widely recognized for their simplicity, cost-efficiency, and fast results, making them ideal for POC testing [15]. By utilizing fluorescent labels purchased from Proteintech (Illkirch-Graffenstaden, France) in CRP detection within an LFIA purchased from DocCheck (Cologne, Germany), the assay’s sensitivity is notably increased compared to gold nanoparticles, offering enhanced precision and reliability for CRP measurement. This dual labeling approach allows for more precise and rapid diagnostics, which is essential in clinical settings.

Fluorescent detection offers higher sensitivity than gold nanoparticles but requires complex optical systems and careful calibration, limiting its usability in POC settings. In contrast, AuNP detection provides simple, robust, and visually interpretable results, making it more practical for field use, though it lacks sensitivity and quantitative precision.

The process begins with a sample pad purchased from Cytiva (Walldorf, Germany), which is used to test samples such as whole blood, serum, or plasma. The sample pad separates the cells from whole blood, allowing only plasma to pass through. This pad is also pre-treated to ensure the smooth migration of the sample to the conjugate pad, which contains CRP-specific antibodies conjugated either to gold nanoparticles or fluorescent nanoparticles such as quantum dots or fluorescent dyes purchased from Proteintech (France) [16]. As the sample flows through the conjugate pad, these antibodies bind to any CRP present, forming a CRP–antibody complex labeled with either gold nanoparticles or fluorescent markers [17].

The sample then continues to the test line, which is coated with a second set of CRP-specific antibodies purchased from Proteintech (France). At this stage, the CRP–antibody complex (tagged with either gold nanoparticles or fluorescent labels) binds to the immobilized antibodies, resulting in the accumulation of signals at the test line [18]. In the case of fluorescent labeling, the intensity of the fluorescence signal will increase if CRP is present, while gold nanoparticles provide a visible colorimetric change [19].

As the fluid moves towards the absorbent pad purchased from Cytiva (Germany) by capillary action, the fluorescence at the test line can be detected and quantified with remarkable speed. The fluorescence intensity (in the case of fluorescent labels) or color intensity (for gold nanoparticles) exhibits a direct correlation with the CRP concentration in the sample, serving as a quantifiable indicator of the biomarker levels. For analysis, the gold nanoparticle-based LFIA employs an 8-bit CMOS Olympus imaging system (Tokyo, Japan), while the fluorescent label-based LFIA uses a 16-bit CCD Olympus imaging system. A fluorescence reader is employed for the quantitative analysis of fluorescent signals, whereas gold nanoparticle detection can be analyzed either through simple visual inspection or imaging, enabling the rapid and efficient determination of CRP concentrations.

Incorporating fluorescence into the LFIA enhances the assay’s specificity because fluorescence signals are inherently less prone to interference from non-specific background signals compared to colorimetric detection [20]. Fluorescence-based detection relies on the excitation and emission of specific wavelengths of light, allowing the system to filter out irrelevant noise and focus on the target signal with higher precision. This reduces the likelihood of false positives caused by non-specific binding or other optical artifacts. Consequently, the improved signal-to-noise ratio provided by fluorescence ensures that only the intended CRP–antibody interactions are measured, thereby increasing both sensitivity and specificity [21]. Advanced imaging systems, such as CCD cameras, further support this specificity, which enhances resolution.

### 2.2. Microfabrication Technology

The microfluidic chip, an essential component in blood cell analysis, is precisely engineered for efficient blood processing [22]. Designed in AutoCAD 2024, it incorporates a blood plasma separation membrane and a capillary pump region with micropillars.

To fabricate the master structure, photolithography was employed [23]. The AutoCAD design was transferred onto a photomask, followed by UV exposure to initiate photopolymerization in the fluidic pathway regions on a silicon wafer coated with SU-8 negative epoxy photoresist purchased from Kayaku (Frankfurt, Germany) [24].

A uniform 30 μm layer of SU-8 photoresist was applied to the wafer via spin coating, and was then subjected to gentle heating at 95 °C for 4 min to evaporate solvents, solidifying the layer. After UV exposure through the channel geometry mask, the photoresist underwent development, with post-exposure heating at 95 °C for 5 min to cross-link the designated geometry. The unexposed areas were removed using a developer solution purchased from Kayaku (Germany), followed by an isopropanol rinse (IPA) and a final intense heating at 150 °C for 30 min to enhance structural durability [25].

A second photolithography stage was required for the thicker, 400 μm double-layer structure, created with a more viscous SU-8 epoxy resin (GM1075-2021) purchased from Kayaku (Germany). Spin speed and UV exposure parameters were precisely adjusted to achieve the intended thickness for the blood plasma separation membrane [26].

The fabricated SU-8 structure on the silicon wafer served as the casting master. To achieve hydrophilic surfaces with a contact angle of less than 2 degrees, 1% polyethylene oxide (PEO) was mixed with the PDMS purchased from Analytics (Bern, Switzerland) before degassing in a vacuum chamber [2,25]. The PDMS–PEO mixture was poured into tSwitzerlandhe casting tray containing the silicon master and cured at 80 °C for an hour. Once cured, the microchip was carefully cut to the desired shape using a surgical blade, ensuring accurate structural replication.

Glass microscope coverslips were then adhered to the top of each PDMS chip, enclosing the microchannels and creating a hydrophilic surface on the top channel [27] while maintaining open inlets and outlets for efficient airflow. This process yields a PDMS microfluidic chip with the precise architectural features necessary for high-accuracy blood analysis.

## 3. Results

This paper presents two distinct designs for the optical detection of CRP. The first design utilizes gold nanoparticles as the detection method, offering a simple and effective approach for visual readouts [28]. The second design incorporates fluorescent labels, which enhance sensitivity by increasing the signal received from the fluorescent labels compared to the gold nanoparticles at the same concentration and also broaden the CRP detection range.

### 3.1. CRP Detection Using Gold Nanoparticles

#### 3.1.1. Microfluidic Chip Design

**Figure 1:**
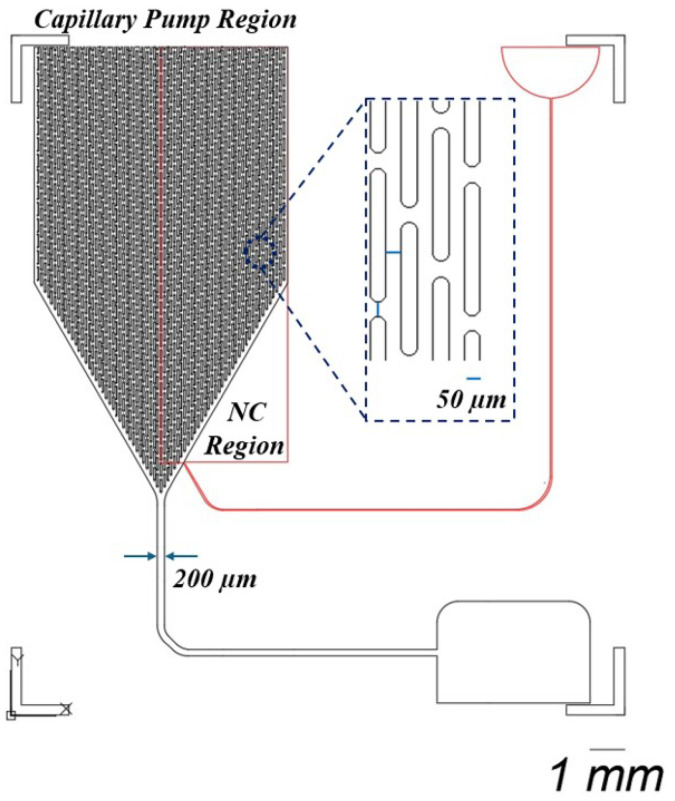
This design consists of two distinct layers. The black layer facilitates microscopic observation of blood samples, incorporating a capillary pump region with micropillars arranged 50 μm apart. This layer is designed with a holding capacity of 2 μL, a channel depth of 30 μm, and a fluidic pathway that measures 200 μm in width. The second layer, depicted in red, contains a nitrocellulose pad (NC) with the test line at a thickness of 400 μm.

As shown in Figure 1, this design integrates two types of detection across two distinct layers. Blood samples are introduced into the chip’s black channel for microscopic observation. This layer also features a CAD-designed layout, which includes a capillary pump region with micropillars spaced 50 μm apart to enhance fluid flow. The first layer of the chip is engineered with a 2 μL holding capacity, a channel depth of 30 μm, and a fluidic pathway 200 μm wide. Potentially, in this channel, blood cells can be observed before the blood is further processed in the capillary pump part.

In the second layer, represented by the red layer in the rectangular region, a nitrocellulose pad containing the test line is placed, incorporating an integrated plasma separation membrane. The porous structure of this membrane facilitates increased fluid flow through the chip, ensuring efficient plasma separation. The second layer has a thickness of 400 μm, providing the necessary structure for fluid dynamics and optimal detection performance.

The reason for using a thicker membrane, which necessitates the fabrication of a second layer, is to facilitate the detection of CRP concentration in the plasma. Typically, a buffer solution is used to push the separated plasma towards the test line [29]. In the red inlet, a buffer solution such as PBS is applied to drive the plasma toward the test line. Additionally, CRP measurement often requires a larger volume of plasma than the first layer, with its 2 μL capacity, can provide, making the second layer essential for accommodating this increased volume.

#### 3.1.2. Image Processing on Microfluidic Chip

**Figure 2:**
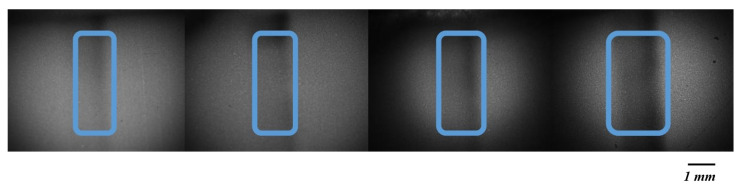
Varying concentrations of CRP on the test line. Images were acquired using a CMOS Olympus imaging system and are in 8-bit format. From left to right, the CRP concentration increases from 5, 10, and 15 to 20 μg/mL. As the concentration increases, the color of the test line becomes progressively darker, indicating a higher intensity.

After the plasma was allowed to pass through the membrane, CRP was facilitated to bind to the antibody conjugated with gold nanoparticles. This process was aided by the use of a buffer solution, which ensured the plasma moved efficiently through the test line. Once this step was completed, the microfluidic chip was positioned under a camera to observe the test line. An image of the test line was captured, as shown in Figure 2, with 20× magnification, ensuring sufficient detail for analysis.

**Figure 3:**
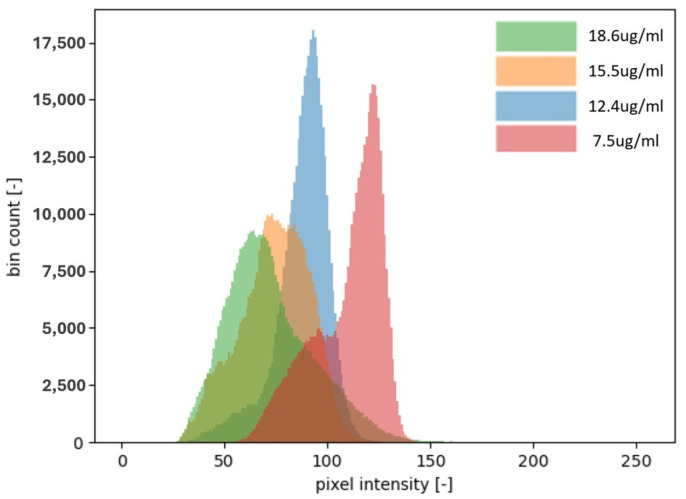
Four different concentrations of CRP and their corresponding intensity distributions on a histogram. The images captured for analysis are 8-bit, meaning the intensity of each pixel is represented by a number between 0 and 255. As shown in the histogram, the higher the concentration of CRP, the more the distribution shifts toward the lower intensity values, becoming closer to 0. This shift indicates a greater presence of black pixels, which correlates with a stronger signal and a higher CRP concentration. In this context, an intensity closer to 0 represents a darker shade, signifying a higher concentration of CRP. Notably, the concentration of 18.6 μg/mL is shifted more to the left compared to the lower concentrations, further illustrating the trend of higher CRP levels producing more intense black signals.

As shown in Figure 3, the analysis of the captured image was carried out using MATLAB software 2024. A region of interest (ROI) was defined first, with the selection focusing on the area exhibiting the highest intensity. Following the identification of the ROI, the intensity of the black color within the test line was measured. The intensity of the pixels was evaluated on a scale ranging from 0, representing black, to 255, representing white [30]. The presence of black color was interpreted as an indication of CRP concentration, with a lower pixel intensity closer to 0 corresponding to a higher concentration of CRP in the sample [31]. Consequently, the darker the test line appeared, the greater the concentration of CRP was determined to be in the plasma [32].

**Figure 4:**
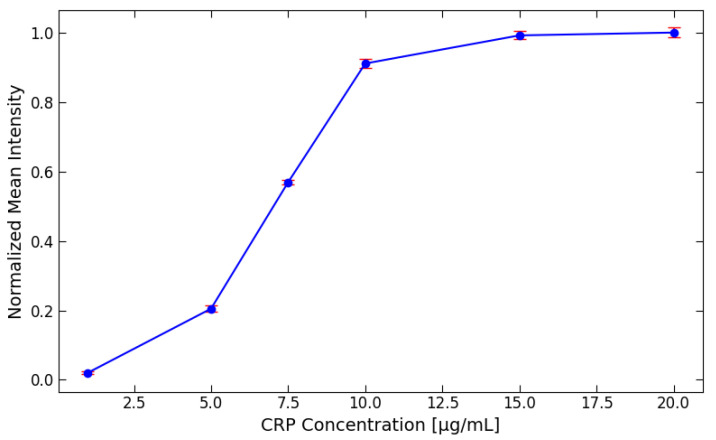
The detection of the CRP concentration using gold nanoparticles at various concentrations (1, 5, 7.5, 10, 15, and 20 μg/mL). After capturing images of the CRP test lines, the signal intensities were analyzed and normalized for comparison.

For the detection of the CRP concentration using gold nanoparticles, different concentrations were employed, as shown in Figure 4. The tested concentrations were 1, 5, 7.5, 10, 15, and 20 μg/mL. After the CRP test line images were captured, the intensity of the signals was analyzed and normalized. The results indicate that the optimal detection range for CRP using gold nanoparticles lies between 1 to 10 μg/mL. Beyond this range, specifically above 10 μg/mL, no significant differences in normalized intensity were observed, suggesting that 10 μg/mL represents the detection limit for CRP with gold nanoparticles. Despite this detection limit, higher concentrations of CRP, up to 100 μg/mL, can still be relevant for healthcare professionals [33], as they can provide important clinical insights into elevated CRP levels. To address the limitations encountered in CRP detection using gold nanoparticles, it was decided to implement green fluorescent labels as an alternative detection method.

### 3.2. CRP Detection Using Fluorescent Labels

#### 3.2.1. Microfluidic Chip Design

Instead of employing various types of membranes to develop an LFIA, the decision has been made to employ a microfluidic chip design due to its distinct advantages over the traditional LFIA structure. One of the primary benefits of using a microfluidic chip is the superior control over fluid dynamics [34]. In a microfluidic chip, fluid flow can be regulated more precisely, allowing it to either proceed through a channel or pause in a specific location. The duration for which the fluid remains in a designated area can also be controlled. This level of control is not achievable with paper-based LFIAs, as capillary action in their three-dimensional structures causes the fluid to flow until it eventually stops, either due to the length of the strip or an insufficient solution. In contrast, the channels within a microfluidic chip can be designed to function effectively even with low fluid volumes, such as 5 μL.

**Figure 5:**
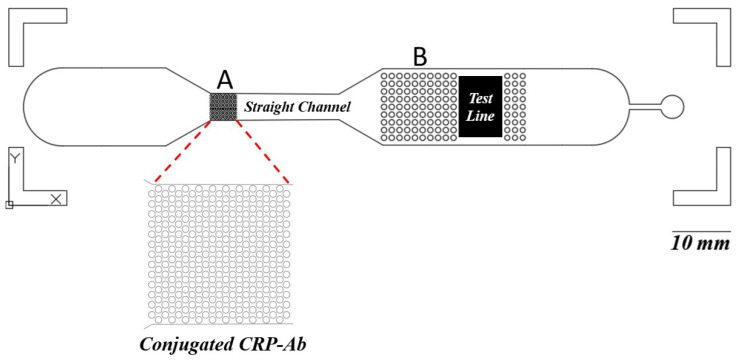
This microfluidic chip allows for the precise regulation of fluid flow and can handle low volumes, unlike paper-based LFIAs, where capillary action limits control. Within the microfluidic device, the component labeled “A” is engineered to facilitate the integration of a conjugate pad post-fabrication. This design balances the necessity for a separate conjugate pad, allowing a drop of antibody solution containing the label to be directly applied and dry onto the designated site. Additionally, this component enhances the consistency of the interaction between CRP in the blood and the conjugated antibodies. Following this interaction, the solution is channeled through a linear pathway, where the length of the channel is critical in determining the duration required for optimal mixing and interaction between CRP and the antibodies. Furthermore, the “B” structures are designed to ensure that the entire NC pad makes uniform contact with the solution, promoting an even distribution across the pad’s surface. This results in a consistent fluorescent signal from the entire NC pad.

Another significant advantage of this microfluidic chip design lies in its ability to incorporate specific structures within the channels that aid in controlling the mixing of blood plasma with antibodies conjugated to fluorescent labels. Proper mixing control is difficult to achieve in paper-based LFIAs due to their inherent design limitations [35]. In the microfluidic chip, as illustrated in Figure 5, the structure labeled “A” facilitates the integration of conjugate pad after the fabrication of the microfluidic chip. Typically, a conjugate pad is required to hold CRP antibodies conjugated with fluorescent labels. To eliminate the need for a separate conjugate pad, the labeled structure is designed to serve as a site where a drop of antibody solution containing the label can be directly applied and left to dry, similar to the conjugate pad, but still with a slightly smaller surface area. The three-dimensional structure labeled “A” significantly increases the available surface area for drying, thereby improving the efficiency of antibody conjugation with the fluorescent labels compared to scenarios where no such structure is present. The additional function of the structure labeled “A” ensures that the mixing of CRP in the blood with the conjugated antibodies is more efficient and consistent. Once this mixing occurs, the solution is directed through a straight channel. The length of this channel plays a crucial role in defining the time required for proper mixing and interaction between CRP and the antibodies.

The straight channel, devoid of any internal structures, provides an extended reaction time for the mixture, thereby enhancing the binding interactions. To ensure the proper binding of CRP to the antibody conjugated with fluorescent labels, several experiments were conducted to optimize the binding time and determine the appropriate flow path length. Initially, an NC pad with dimensions of 0.5 cm in width and 1.5 cm in length was used for CRP detection. However, it was observed that the binding efficiency was suboptimal. Consequently, the pad length was progressively increased to 2, 2.5, and 3 cm. The results indicated minimal differences in the binding efficiency between 2.5 and 3 cm. Therefore, a straight channel with a length of 4 cm was designed to provide additional time for the reaction, thereby improving the binding efficiency. The increased reaction efficiency subsequently leads to the improved sensitivity of the detection system.

Extending the length of the NC pad increases the required liquid volume, which consequently raises the volume requirements for both sample- and buffer-washing steps. In contrast, microfluidic systems allow for the design of smaller channels, which reduces the overall volume while still enabling extended reaction times by increasing the channel length.

When the LIFA was not integrated with microfluidic technology, increasing the length of the NC pad resulted in a significant cost increase. This was not only due to the increased use of NC membranes but also because of the additional reagents, such as BSA for blocking, which are expensive. However, the integration of microfluidic technology enables a one-step detection system, eliminating the need for buffer solution steps and significantly reducing the NC pad area to only include the detection test line. This approach minimizes the non-specific binding of conjugated antibodies to unwanted areas of the NC pad, thereby eliminating the need to block those regions. By directing the reagent flow specifically to the detection line and reducing interference from other areas, this method significantly enhances detection sensitivity, leading to more accurate and reliable results.

Moreover, the structures labeled “B” are designed to ensure that the entire NC pad comes into uniform contact with the solution. This ensures that the solution is evenly distributed across the NC pad, thereby allowing the entire surface of the pad to emit a consistent fluorescent signal. Achieving this level of uniformity in fluorescent intensity is challenging with paper-based LFIAs, where the uneven distribution of the solution often leads to inconsistent results. The use of microfluidic chips thus provides a more reliable platform for immunoassays by offering enhanced control over fluid flow, mixing, and distribution—features that are not easily achievable in traditional LFIA systems.

#### 3.2.2. Fabrication of Test Line

To utilize fluorescence detection in our experiment, we selected the CoraLite^®^ Plus 488-conjugated CRP Monoclonal antibody. This antibody has excitation and emission maxima at 493 nm and 522 nm, respectively [36], which means that green fluorescent labels are employed for detecting CRP. A crucial component in the detection process is the NC pad, which plays a significant role in ensuring that the antibody binds effectively during the assay.

The porous structure of the NC pad is a critical factor. It must allow for the physical adsorption (physisorption) and mechanical bonding between the antibody and the pad. This binding needs to be strong enough to withstand the washing steps, so that the antibody remains firmly fixed at the test line. Taking this into account, a specialized NC pad is opted for, specifically the FF120HP, which is well suited for this type of application.

However, one of the disadvantages of using the FF120HP NC pad is that it can exhibit a green background color [37], which can interfere with the fluorescence detection. This phenomenon is commonly referred to as the “background effect”. The background effect occurs when the material or substrate used in the detection system produces fluorescence or absorbs light at wavelengths similar to the fluorescent label. In this case, the green tint of the NC pad can overlap with the green fluorescence emitted by the CoraLite^®^ Plus 488-conjugated antibody, thereby reducing the signal-to-noise ratio. This interference can make it more challenging to accurately distinguish the specific signal related to CRP detection from the background fluorescence of the NC pad itself.

**Figure 6:**
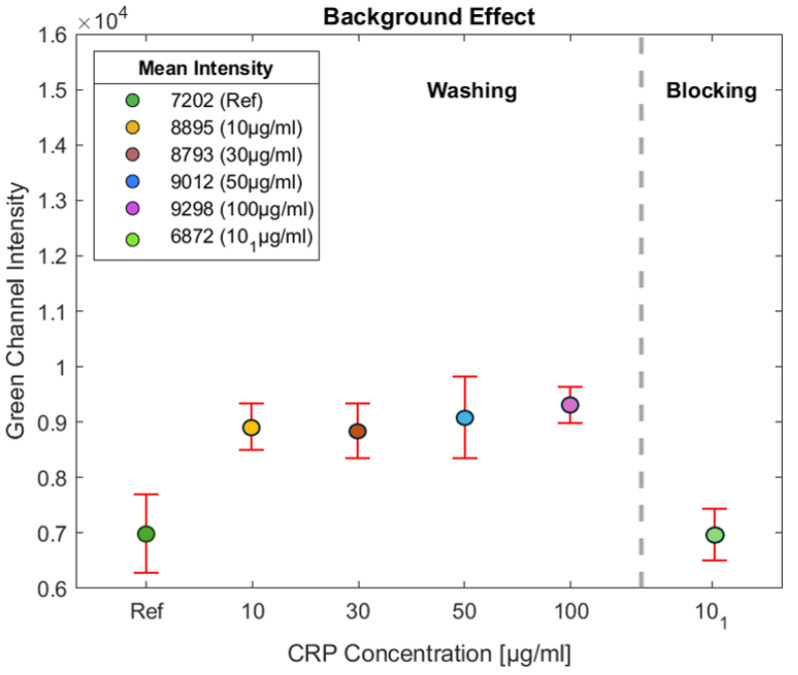
Several experiments were performed to assess the background effect of the NC pad. The mean background fluorescence intensity was measured at 7202, indicating the detection limit for the pad. Tests with CRP concentrations of 10, 30, 50, and 100 μg/mL were also conducted to evaluate the effect of BSA on detection. The green light intensity measured outside the test line on the NC membrane remained consistent at approximately 9000, indicating elevated non-specific binding. Comparison with the reference NC shows an increase of about 1800 units, demonstrating BSA’s washing role in reducing non-specific binding in the assay.

As shown in Figure 6, several experiments were conducted to investigate the background effect of the NC pad. The background effect refers to the inherent fluorescence or signal produced by the NC pad, which can interfere with the accurate detection of the target signal. After analyzing the results, it was determined that the mean intensity value of the background signal generated by the NC pad was 7202. This value represents the detection limit for the NC pad, indicating that any fluorescence signal below this value cannot be accurately measured, as it is obscured by the background fluorescence of the NC.

This threshold is used as a reference point for the fluorescent detection system in the green channel, which is essential for identifying the presence of the target analyte—in this case, CRP. Fluorescence signals with intensity values lower than this limit may not be distinguishable from the NC background. Therefore, this effect must be accounted for during data analysis. By recognizing the background threshold, it is ensured that any fluorescence intensity measured above 7202 can be attributed to the actual presence of CRP rather than the NC pad. This understanding is crucial for the accurate interpretation of the fluorescence detection results, highlighting the importance of optimizing experimental conditions to reduce background interference.

#### 3.2.3. Controlling Non-Specific Binding

Non-specific binding is a frequent challenge in lateral flow assays where components from the sample or the detector conjugates bind to the membrane surface outside the designated test and control lines. This issue compromises the test’s accuracy, as target analytes can be lost or misinterpreted. The impact of non-specific binding is significant. It can result in false positives, where the assay incorrectly indicates the presence of the target, or false negatives, where the target is present but not detected. Such errors are particularly concerning in critical medical applications, where accurate diagnosis is essential.

To address non-specific binding, two main strategies are used. The first involves applying a blocking agent (such as proteins, polymers, or surfactants) to the membrane during manufacturing, covering free binding sites and ensuring only the test and control lines capture the target analytes. This approach reduces non-specific binding and creates a controlled environment. It is cost- and time-efficient by minimizing reagent use and fine-tuning membrane properties, but it requires an extra manufacturing step and may lead to variability in the amount of blocking agent applied.

The second approach, known as “blocking on the fly”, involves incorporating the blocking agent into the sample pad. As the sample flows through the lateral flow test, the blocking agent is released and actively blocks open binding sites outside the test and control lines in real time. This dynamic blocking process helps prevent non-specific binding as the assay progresses.

While blocking on the fly can be integrated into the conjugate pad manufacturing process, it presents challenges, such as the potential for high surfactant concentrations near the test line, which can disrupt proper interactions between the target analyte and the test line. Additionally, this method can introduce inconsistencies in the thickness of both the conjugate pad and the membrane, leading to variability in the flow rates and overall assay performance.

In light of the advantages mentioned above, Bovine Serum Albumin (BSA) was selected as the blocking agent for the lateral flow assay. BSA is widely known for its ability to prevent non-specific binding, although its concentration significantly influences the assay’s overall performance. During the experiments, solutions containing BSA in PBS (phosphate-buffered saline) with 0.1% Tween 20 were prepared, and the BSA concentration was varied to determine the optimal condition.

Initially, a solution with 0.5% BSA was prepared, but it was soon recognized that adjusting the concentration could improve performance. To explore this, the BSA concentration was increased to 1% and subsequently to 1.5%, while the effects on the blocking process were observed. Through these trials, it was determined that 1.5% BSA provided the most effective blocking. At this concentration, BSA successfully prevented the non-specific binding of the fluorescently conjugated antibody, allowing it to travel through the nitrocellulose membrane without premature immobilization.

The 1.5% BSA concentration created a more robust blocking layer, preventing the fluorescently labeled antibody from adhering to unintended areas of the membrane before reaching the test line. This improvement enhanced the accuracy and consistency of the assay by ensuring the proper immobilization of the antibody–fluorescent conjugates at the test line, where the target analyte (CRP) would be detected. In contrast, lower BSA concentrations, such as 0.5% or 1%, provided insufficient blocking, leading to the partial fixation of the antibody conjugates in undesired regions of the membrane.

In Figure 6, tests were conducted with CRP concentrations of 10, 30, 50, and 100 μg/mL. The intensity of the green light was measured on the NC membrane outside the test line to assess the effect of BSA on detection. As observed, for all experiments, the intensity outside the test line remains consistent at approximately 9000, indicating a higher level of non-specific binding. By comparing this intensity with the reference (NC without BSA), it is evident that the impact of BSA results in an increase of around 1800 units, demonstrating its role in minimizing non-specific binding in the assay.

As illustrated in Figure 6, experiments were conducted using a CRP concentration of 10 μg/mL, labeled as $10_{1}$, to investigate the effect of BSA on the NC pad. This approach aims to minimize the intensity of BSA on the NC pad. Initially, CRP antibodies were applied to the NC pad at a concentration of 1 mg/mL, with a volume of 1.5 μL, resulting in a total of 1.5 μg of CRP antibodies deposited on the test line. This application was performed using a micropipette, and the test line was incubated at 37 °C for 25 min.

Following this step, the NC pad was immersed in a BSA solution at a concentration of 1.5% to allow the BSA to permeate through the porous structure of the NC pad. The NC pad was then placed in an oven at 37 °C for 60 min to facilitate drying. The experimental results indicate a significant reduction in the background effect, correlating with a decrease in the intensity of the green channel on the NC pad. This demonstrates the effectiveness of the employed method in mitigating the background fluorescence, thereby enhancing the accuracy of the assay.

#### 3.2.4. Image Processing for CRP Detection

The detection of CRP was initiated by applying a specific concentration of CRP onto an NC pad. A BSA solution was then added as a blocking agent, and the solution was allowed to migrate toward the test line. After the solution reached the test line, a washing step was performed to remove any unbound substances.

**Figure 7:**
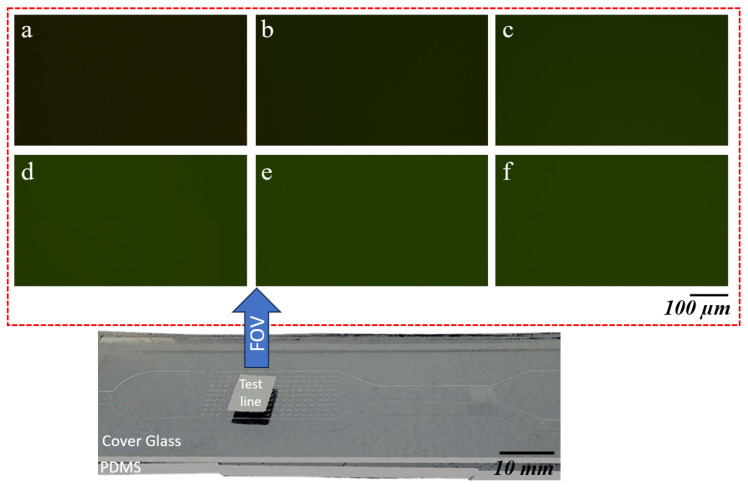
Panels (**a**–**f**) display the green channel intensity images captured under 20× magnification for varying concentrations of CRP on an NC pad. Starting with the reference sample (**a**) at 0 μg/mL CRP concentration, the intensity increases as the CRP concentration rises. Panel (**a**) shows an intensity of 7095, followed by (**b**) 9108 for 10 μg/mL, (**c**) 12,401 for 30 μg/mL, (**d**) 14,692 for 50 μg/mL, (**e**) 15,602 for 70 μg/mL, and (**f**) 15,262 for 100 μg/mL CRP concentration. The increase in intensity correlates with the increasing CRP concentrations applied to the NC pad.

The test line was subsequently placed under a microscope connected to a camera for imaging. An initial magnification of 4× was used to locate the area of the test line with the highest intensity. Once identified, the magnification was increased to 20× to capture a detailed image of the region exhibiting the highest intensity, as shown in Figure 7. The figure illustrates images captured for different CRP concentrations, ranging from a reference sample with no concentrations of CRP to samples with CRP concentrations of 10, 30, 50, 70, and 100 μg/mL. In the reference sample, which contains no CRP, the intensity shown corresponds solely to the green channel of the NC pad.

As the CRP concentration increases, a clear trend can be observed. The intensity in the green channel progressively grows with higher concentrations of CRP. This increasing intensity indicates that as more CRP is present in the sample, the signal becomes stronger, resulting in a lighter appearance of the green channel in the corresponding images. The visual difference in intensity is especially pronounced at higher concentrations, where the green hue becomes significantly lighter compared to the reference sample.

**Figure 8:**
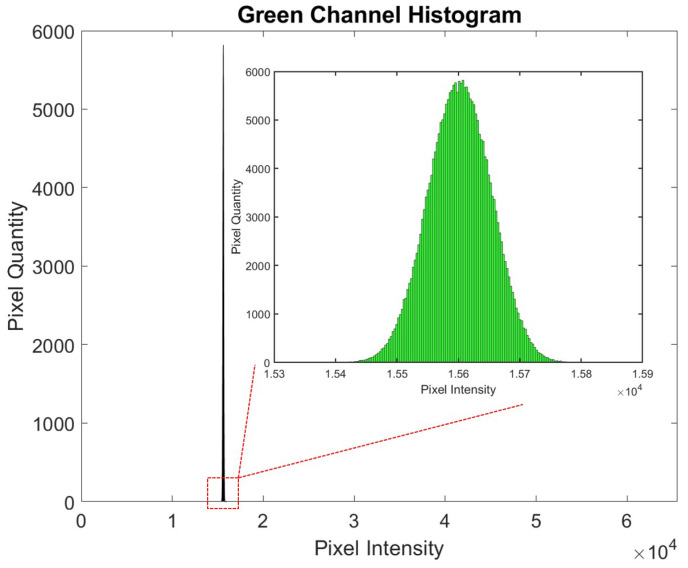
Histogram showing pixel intensity values plotted against the pixel quantity. The images were captured in a 16-bit format, allowing intensity values to range from 0 to 65,535, with higher values representing greater intensity.

A MATLAB code was developed for image analysis. The code was programmed to define an ROI within the image, focusing on the area with the strongest signal. The mean intensity of the selected channel within the ROI was then calculated, providing a quantitative measure of the signal, as depicted in the corresponding Figure 8. The histogram displays pixel intensity values plotted against the pixel quantity. Since the images were captured with a 16-bit format, the intensity values can range between 0 and 65,535, with higher values indicating greater intensity.

As the CRP concentration increases, the corresponding signal becomes lighter, resulting in a rise in pixel intensity, a trend consistent with the characteristics of fluorescent labeling. In contrast, gold nanoparticle detection demonstrates an inverse relationship, where signal intensity decreases as the CRP concentration increases.

**Figure 9:**
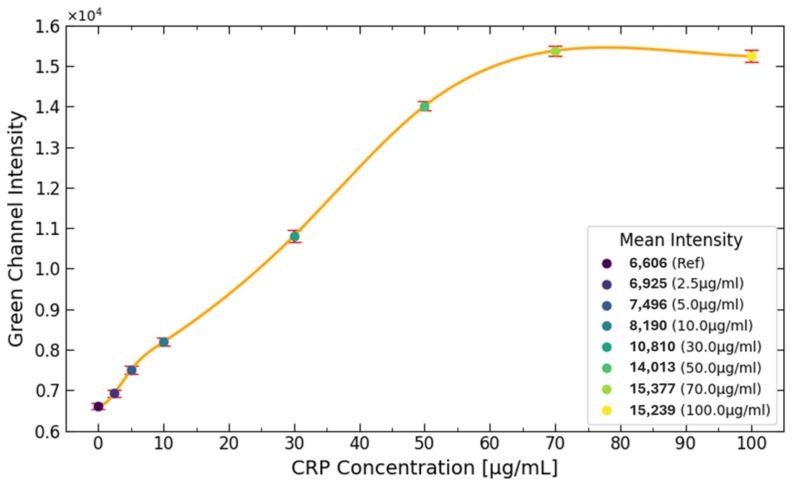
The diagram illustrates the effect of different CRP concentrations on signal intensity. It is evident that the CRP concentration increases steadily from 2.5 to 70 μg/mL. However, once the concentration reaches 70 μg/mL, the signal plateaus, indicating that the upper detection limit has been achieved. This plateau is further supported by the mean intensity values of the 70 and 100 μg/mL samples, which fall within the same range.

Different CRP concentrations were used to generate the diagram shown in Figure 9. It can be observed that the pixel intensity increases steadily between 2.5 to 70 μg/mL. However, after reaching 70 μg/mL, the signal plateaus, indicating that the upper saturation limit has been reached. This is evident from the mean intensity values of the 70 and 100 μg/mL samples, which fall within the same range.

The reason for this plateau is due to the limited number of antibodies present on the test line. These antibodies, which bind to the CRP antibody conjugated with fluorescent labels, become fully saturated as the CRP concentration reaches the upper limit. The number of antibodies available for binding on the test line is influenced by the porous structure of the NC pad. In this case, the NC pad can only accommodate around 1.5 μg of CRP on the test line, preventing further binding and signal increasing beyond this point.

### 3.3. Comparative Analysis of Histogram Patterns

A distinct difference in the histogram shapes between Figure 3 and Figure 8 can be observed. Specifically, in the analysis of the CRP concentration with the use of gold nanoparticles, Figure 3 demonstrates a bimodal distribution in most cases, indicating the presence of two distinct peaks. This bimodal nature poses challenges in interpreting a single peak as the characteristic marker for the CRP concentration. Consequently, calculating the mean CRP concentration becomes more complicated, as it is possible that the two peaks may represent different concentrations that yield similar average values. This ambiguity may contribute to the inability to accurately measure CRP concentrations above 10 μg/mL, as depicted in Figure 4.

Another limiting factor affecting CRP quantification beyond 10 μg/mL is the constrained dynamic range of pixel intensity, which spans from 0 to 255. This limited intensity range restricts the ability to capture variations in CRP concentrations at higher levels.

Conversely, Figure 8 exhibits a histogram with a single, well-defined peak consistently located near the midpoint of the histogram. Under these conditions, the peak and the mean values of the histogram are nearly identical, facilitating more straightforward analysis. Moreover, the extended pixel intensity range of 0 to 65,535 allows for a broader detection range, enabling reliable measurements of CRP concentrations up to 70 μg/mL without the limitations encountered in the lower-intensity range of earlier measurements.

Although the pixel intensity range is higher, it can be observed that this does not affect the consistency of the histogram shown in Figure 8. The histogram values are consistently within the range of $1.54\;\mathrm{to}\;1.58\times 10^{4}$, showing a Gaussian distribution with a small standard deviation.

## 4. Discussion

Table 1 compares the proposed microfluidic CRP detection methods with conventional LFIAs (AuNP), latex turbidimetric immunoassays, and ELISAs in terms of detection range, sensitivity, assay time, sample volume, instrumentation requirements, and overall complexity. Conventional LFIAs often exhibit limited upper detection ranges (10–50 μg/mL) and reduced sensitivity below 10 μg/mL, while methods like latex turbidimetric immunoassays or ELISAs enhance sensitivity but require specialized equipment and longer processing times. In contrast, the microfluidic AuNP method achieves a high sensitivity range of 1–10 μg/mL, suitable for chronic disease monitoring, and the microfluidic fluorescent platform further extends this detection range to 70 μg/mL, covering higher CRP levels linked to acute infections. Both microfluidic approaches require minimal sample volume (5–10 μL) and shorter assay times (about 5–8 min), mainly because fluid flow is precisely controlled within microchannels, thus circumventing additional buffer solutions. This microfluidic design also localizes the reaction to the test line, reducing non-specific binding and eliminating the need to block extra pad regions. As a result, these microfluidic LFIAs combine an extended detection range with lower reagent usage, enabling cost-effective POCT in resource-limited settings.

Both microfluidic methods offer improvements over conventional LFIAs regarding the LOD and signal-to-noise ratio. The controlled flow rate and reduced non-specific binding enhance the clarity of the detection signal, thereby increasing the signal-to-noise ratio. Environmental factors such as temperature and humidity must still be considered. However, the smaller reaction area in microfluidic formats can sometimes mitigate these effects if the cartridge or chip is well sealed. Concerning portability and power needs, the microfluidic AuNP method retains a visual readout comparable to standard LFAs in low-power settings. The fluorescent method does require a fluorescence detector, but compact battery-powered readers are increasingly available. Regarding regulatory approval and scalability, the simplified one-step format and lower material demands support large-scale manufacturing, which can facilitate broader market entry and potential approval. Finally, from a cost perspective, while fluorescence-based detection may be slightly more expensive due to the reader, microfluidic LFAs overall reduce the per-test reagent volume, making them competitive alternatives to traditional lab-based assays for CRP detection and potentially easing compliance with regulatory and economic constraints.

## 5. Conclusions

In conclusion, this study examines two methods for measuring the concentration of CRP, with one utilizing gold nanoparticles and the other employing CRP antibodies conjugated with green fluorescent labels. Both methods are advantageous in that they do not require expensive machinery for the deposition process. This accessibility means that the techniques can be implemented even in resource-limited areas, facilitating the creation of easy-to-use devices for CRP detection.

The gold nanoparticle-based method demonstrates a detection range between 1 μg/mL and 10 μg/mL. While this range is adequate for certain diagnostic applications, it is relatively narrow. In contrast, the fluorescent label-based method extends the detection range significantly, from 1 μg/mL up to 70 μg/mL. This broader range enhances the method’s versatility and applicability across a wider spectrum of clinical scenarios, where CRP levels can vary substantially among patients.

The proposed design for CRP detection using fluorescent labels is particularly effective due to its integration with microfluidic technology. The microfluidic design allows for greater control over the timing and mixing of CRP with the conjugated antibodies. This level of control over the flow is challenging with conventional lateral flow immunoassays. Enhanced control over these parameters can make the assay more reliable and reproducible.

The fluorescent label-based method, powered by microfluidic design, presents a superior approach for CRP detection. It combines the benefits of a broader detection range with enhanced control over assay conditions, all without the need for expensive equipment. This makes it an ideal solution for improving healthcare outcomes in both well-equipped and resource-constrained environments.

Furthermore, future enhancements could incorporate smartphone-based imaging or AI/ML-driven data processing to automate result interpretation and improve clinical decision-making. Such advancements would minimize operator dependency, reduce subjectivity in result reading, and facilitate real-time diagnostics. Extending the current platform to detect multiple biomarkers simultaneously would transform the system into a multi-analyte device, enabling comprehensive disease monitoring from a single sample. Implementing these strategies would greatly expand the clinical utility of the technology while preserving its accessibility and cost-effectiveness.

## Figures and Tables

**Table 1:** Comparison of the proposed microfluidic CRP detection methods with conventional CRP assays.

Parameter	Conventional LFA (AuNP)	Latex Turbidimetric Immunoassay	ELISA	Microfluidic LFA (AuNP)	Microfluidic LFA (Fluorescent)
Detection Range (μg/mL)	∼10–50	0.2–200 *	0.1–50	1–10	1–70
LOD (μg/mL)	∼10	0.2–0.5 *	0.1 or lower	∼1	∼1
Assay Time (min)	15–20	10–15	120–240	5–8	5–8
Sample Volume (μL)	10–20	10–50	50–100	5–10	5–10
Instrumentation	Minimal (visual)	Bench-top analyzer	Plate reader	Minimal (visual)	Fluorescence detector
Complexity	Low	Moderate (instrument needed)	High (multiple steps)	Moderate (microfluidic integration)	Moderate (microfluidic + reader)
Reagents Required	One-step or two-step buffer	Multiple reagent solutions	Multiple wash buffers	No buffer needed	No buffer needed
Cost	Low–Moderate	Moderate–High	Moderate–High	Low–Moderate	Moderate
Major Advantages	Simple, widely used	Good sensitivity; automated options	High sensitivity, well-established	Rapid, enhanced sensitivity, minimal reagents	Extended detection range, high sensitivity, minimal reagents
Major Drawbacks	Lower sensitivity for CRP < 10 μg/mL	Requires special instrument	Longer assay time, lab-based	Requires microfluidic fabrication	Requires fluorescence reader

* Values may vary depending on commercial kit and instrument settings.

## Data Availability

Data are contained within the article.

## Abbreviations

The following abbreviations are used in this manuscript:

BSA	Bovine Serum Albumin
CRP	C-reactive protein
ELISA	Enzyme-Linked Immunosorbent Assay
FOV	Field of view
IPA	Isopropanol
LFIA	Lateral flow immunoassay
NC	Nitrocellulose
PBS	Phosphate-buffered saline
PDMS	Polydimethylsiloxane
PEO	Polyethylene oxide
POCT	Point-of-care testing
ROI	Region of interest
Int	Intensity
